# Intercurrent Remedies for Chronic Diseases

**Published:** 1889-05

**Authors:** 


### I. INTERCURRENT REMEDIES FOR CHRONIC DISEASES.

[Translated from Dr. C. v. Bœnninghausen's Repertory of Antipsoric Remedies.—F. H. Lutze.]

COFFEA (X°R) *i. e.* ($^{30}$) for over-sensitiveness and painfulness of diseased parts, fretfulness, and sleeplessness.

HEPAR SULPH. CALC. alternately with Nitric acid for over-excitement from abuse of Mercury.

MAGNES. ARCT. for over-excitement with trembling, fidgetiness of the extremities, great distention of abdomen, anxious irresolution; solicitous and great nervous debility.

MESMERISMUS. Nervous debility in general.

NUX VOMICA (X°R.), if the nervous system is too much affected and irritated; hyperæsthesia of the organs of special sense; fearfulness, anxiety, inclination to lie down, aversion to the open air, violent, stubborn, obstinate; also, if the menses appear too early or continue too long.

OPIUM (X°R). Lack of sensitiveness of the nervous system, deficient reaction of the life-force. (*Carbo veg.*, *Laurocer.*, *Mosch.*, *Nitr. ac.* or *Sulph.* (all in X°R.) may also be useful here.)

PULSATILLA (X°R) in some cases, with proper intervals, alternately with Nux v. to remove too great an irritability.

In rare cases, if there exists too great an irritability of the nervous system; Asarum, Chamom., China, Ignat., Teucrium, or Valeriana may have to be used in the same manner, if these remedies correspond better to the general condition.

### II. REMEDIES FOR DISTURBANCES OF THE ANTIPSORIC CURE.

Bruises and wounds: Arnica X°R.

Burns, superficial: repeated applications of hot alcohol or oil of turpentine.*

Cold, catching of, in general: Nux vom. X°R.
followed by attacks of dyspnœa, Ipecac. III°R.
followed by catarrh, with loss of smell and taste: Puls. X°R.
diarrhœa: Dulcam. X°R.
fever and heat: Acon. X°R.
pain and inclination to weep: Coffea X°R.

Debility, from loss of fluids; as sweat and pollutions, etc.: China X°R.

Fright, causing fear (immediately after): Opium X°R.
followed by grief: Ignatia X°R.
with vexation: Acon. X°R.

Homesickness, with red cheeks and sleeplessness at night: Capsicum X°R.

Inebriation, bad effects of, from wine, etc.: Nux v. X°R.

Love, unhappy with quiet grief: Ignatia X°R.
jealousy: Hyos. X°R.

Protrusion of hernia; most generally: Nux v. X°R.

Stomach, chilling of, as per example, with fruits, etc.,: Ars. X°R, or sometimes Puls.$^{30}$.
deranged, from fat, especially pork: Puls. X°R.
with regurgitation of what has been eaten, nausea and vomiting: Antimon. crud. X°R.
with gastric fever, chilliness and coldness: Bry. X°R.
overloaded: abstinence and drinking a little coffee.

Sprains and over-lifting, effects of: in some cases Arnica, but better: Rhus tox. X°R.

Vexation, with anger, violence and heat: Chamom. X°R.
causing quiet anger, grief, or shame: Ignat. X°R.
with fretfulness, and accompanied with chilliness and coldness of the body: Bryonia X°R.
indignation and throwing away of whatever one holds in his hands: Staphisagria X°R.

---

* Cantharis low externally and high internally. Transl.

